# A novel method to prepare tussah/*Bombyx mori* silk fibroin-based films

**DOI:** 10.1039/c8ra03266a

**Published:** 2018-06-15

**Authors:** Richeng Yang, Peng Wu, Xinhong Wang, Zekun Liu, Cong Zhang, Yinglu Shi, Feng Zhang, Baoqi Zuo

**Affiliations:** National Engineering Laboratory for Modern Silk, College of Textile and Clothing Engineering, Soochow University Suzhou 215123 China bqzuo@suda.edu.cn; Department of Orthopedics, The Second Affiliated Hospital of Soochow University Suzhou 215006 China; Medical College of Soochow University, Key Laboratory of Stem Cells and Biomedical Materials of Jiangsu Province and Chinese Ministry of Science and Technology Suzhou 215123 China fzhang@suda.edu.cn

## Abstract

The possibility of using silk fibroin in biomaterials for tissue engineering is a subject of broad interest. In this study, *Bombyx mori*/tussah silk fibroin (BSF/TSF) blend films were prepared by solution casting using CaCl_2_/formic acid as a co-solvent and water as a rinse solvent. The morphology, crystallinity, thermal resistance, mechanical properties and water contact angle of the blend films as well as the biocompatibility were investigated. The BSF/TSF blend films displayed a smooth surface and specific nanostructure in their cross-section, originating from the nanofibril-preservation during fibroin dissolution. The water rinse process induced the formation of a stable β-sheet structure. The BSF film showed superior mechanical properties to the TSF film, and the blending with TSF led to a significant reduction in the strength and elasticity of blend films. However, adding the TSF component could regulate the hydrophilic properties and enhance cell growth on the blend films. The BSF/TSF blend films with specific nanostructure, stable secondary structure, appropriate mechanical properties, and good biocompatibility, are promising candidates for application in regenerative medicine.

## Abstract 2

A novel method is reported to prepare tussah/*Bombyx mori* silk fibroin blend films featured transparent, flexible and biocompatible.

## Graphical abstract

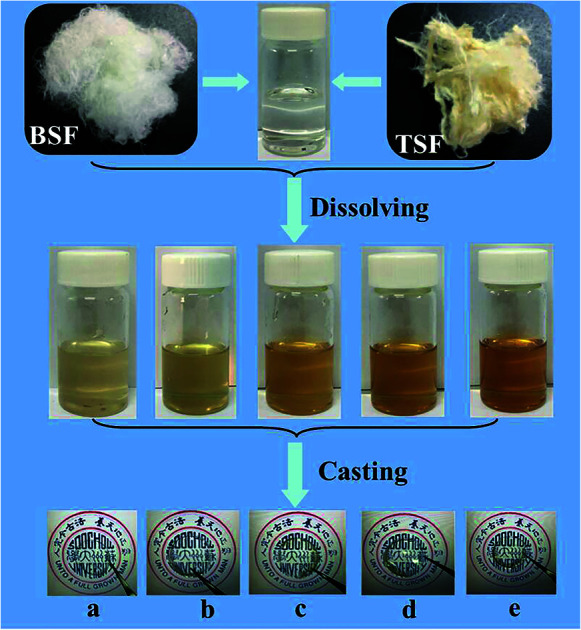

## Introduction

Silk is a natural polymer produced by domestic and wild silkworms, which has been used in the textile industry for thousands of years.^1,2^ Recently, silk fibroin has been widely studied for potential application in biomedical and biotechnological areas because of its excellent mechanical properties, biological compatibility, and biodegradation.^3,4^*Bombyx mori* silk fibroin (BSF), the most investigated fibroin-based biomaterial, has been processed into various forms for engineering of bone, vascular, neural, skin, cartilage, ligaments, cardiac and bladder tissue.^5^ Although giving generally promising results in terms of cell adhesion and proliferation, and tissue growth, the ability of BSF materials to promote early cell adhesion is insufficient due to the lack of specific functional molecules.^6^ The incorporation of the Arg-Gly-Asp (RGD) sequence, a cell-attachment recognition signal, has been a widely used strategy to improve the cell-adhesive properties of BSF.^7^

Compared with BSF, tussah silk fibroin (TSF) molecule inherently contains the RGD sequence and more amino acids with positive charge which favors cell adhesion.^8^ Therefore, blending BSF and TSF is a potential strategy to prepare silk fibroin-based material with excellent mechanical properties and improve biocompatibility. To prepare BSF/TSF blend films, the dissolution of BSF and TSF are required. A series of solvents has been successfully developed to dissolve BSF, such as aqueous lithium salt solution, CaCl_2_/ethanol/water solution.^9^ Although the prior solvents is excellent solvent for BSF, TSF is insoluble in these solvents.^10^ To date, only limited work on TSF dissolution and regeneration has been reported.^11^ TSF has been reported soluble in melt Ca(NO_3_)_2_ at 105 °C,^12,13^ LiSCN at 40–55 °C,^14^ and ionic liquid.^11^ In addition, TSF has been extracted directly from the glands of silkworms.^15–17^ The as-cast BSF and TSF films are water soluble due to their random coil structure, post-treatment using organic solvents is required, such as methanol, ethanol.^18,19^ To develop highly biocompatible silk fibroin material, using less toxic solvents is an important direction.^20^ Therefore, the main problem of the present silk fibroin dissolution and water-insoluble film preparation is complicated and time-consuming, and is difficult to scale up, and the employment of toxic solvent.

Recently, we showed that CaCl_2_/formic acid could serve as new dissolving solvent for BSF, and water as post-treatment solvent directly.^21^ In this study, we employed CaCl_2_/formic acid as a co-solvent to dissolve BSF and TSF at room temperature, and to prepare BSF/TSF blend films using water as rinse solvent. We evaluated and discussed here the morphology, structure, thermal stability, and mechanical properties using a combination of SEM, FTIR, XRD, TGA and DSC, and mechanical testing of films composed of BSF, TSF and their blends, as well as the films' biocompatibility using cell culture experiment.

## Experimental methods

### Materials

*Bombyx mori* silk fibers and tussah silk fibers were purchased from Zhejiang Province and Liaoning Province, China, respectively. All chemical regents (calcium chloride, formic acid, *etc.*) were bought from Sinopharm Chemical Reagent Co., Ltd. (Shanghai, China), and used without any further purification.

### Preparation of BSF and TSF blend films

**Fig. 1:**
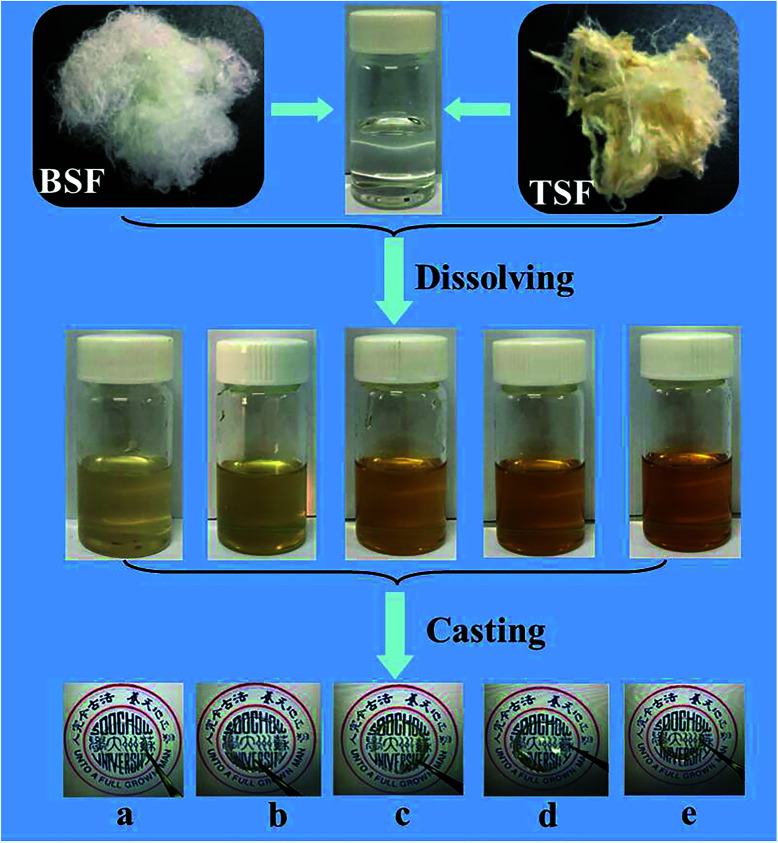
Preparation process of BSF/TSF blend films with various blend ratios. (a) BSF, (b) 3/1, (c) 1/1, (d) 1/3, (e) TSF.

The preparation process of BSF/TSF blend films were illustrated in Fig. 1. The degumming process of silk was conducted according to our previous published procedures.^22^ Raw *Bombyx mori* silk fibers were boiled in 0.05 wt% Na_2_CO_3_ solution for 30 min and rinsed thoroughly with deionized water to extract the glue-like sericin proteins. In addition, tussah silk fibers were boiled in 0.5 wt% Na_2_CO_3_ solution for 30 min and rinsed thoroughly with deionized water to extract the glue-like sericin proteins. The above steps was repeated thrice, then the degummed silk fibers were dried at room temperature. After drying, the degummed BSF and TSF were dissolved in 10% CaCl_2_–formic acid solution at room temperature with ratio of 1/3, 1/1 and 3/1. The mixture solution was cast upon polystyrene dishes (diameter 90 mm) for drying. The as-cast films were immersed in deionized water to remove salt ions. After drying, the desired BSF, BSF/TSF blend and TSF films were prepared. The schematic process for film preparation was showed in Fig. 1.

### SEM imaging

The surface and cross-section of blend films were observed with S-4800 SEM (Hitachi, Tokyo, Japan) at 3 kV. For cross-section imaging, the films were fractured in liquid nitrogen to avoid deformation.

### FTIR analysis

The structure of blend films prepared by CaCl_2_–formic acid dissolution method were analyzed by FTIR on Nicolet 5700 (Thermal Nicolet Company, USA) in transmittance mode. For each measurement, each spectrum was obtained by the performance of 32 scans with the wavenumber ranging from 400 to 4000 cm^−1^ with a resolution of 4 cm^−1^.

### X-ray diffraction (XRD)

X-ray diffraction experiments were also measured on X Pert-Pro MPD (PANalytical, Netherlands) in transmittance mode to investigate the crystalline structure of samples. The incident beam wavelength was 0.154 nm. The intensity was finally corrected for changes in the incident beam intensity, sample absorption, and background.

### Thermogravimetric measurements

Thermogravimetry/differential thermal analysis (TGA-DTA, PE-SII, America) condition were nitrogen flux at 30 ml min^−1^, heating rate at 10 °C min^−1^ and temperature range from 50 to 600 °C, and the samples' weight was about 5 mg.

### Mechanical properties

Blend films samples, prepared similar as above, were cut into 50 mm × 10 mm rectangle with thickness of 100–120 μm. The micrometer was used to measure thickness of these samples. Before using an automatic tensile tester (model 3365 electronic strength tester, Instron, Boston, USA) to investigate the mechanical properties of films, these films were kept for 24 hours at atmospheric conditions (20 °C and 65 RH). During test process, distance between grips and test speeds were set to 20 mm and 10 mm min^−1^, respectively. At the same time, the pre-tension was 0.2 cN. An average of twenty measurements was reported as the mean ± standard deviation for each sample. The formulas for breaking strength and extension at break were as follows:Extension at break (%) = specimen elongation (mm)/original length (mm) × 100%;Breaking strength (MPa) = breaking force (N)/(specimen thickness (mm) × specimen width (mm))

The linear elastic modulus was calculated using a least squares fitting between 0.1 N load and 2.5% strain past that point. Ultimate tensile strength was the highest stress value attained during the test and the elongation to failure was the last data point before a >10% decrease in load.

### Contact angle

The drop of pure distilled water of volume 5 ml was placed on the film surface using a syringe with a 22-gauge needle. The static contact angle was measured using a Kruss GmbH, Germany. The measurement of each contact angle was made within 10 s after each drop to ensure that the droplet did not soak into the compact. The contact angles reported were the mean of 10 determinations. Smaller contact angles corresponded to increased wettability.

### *In vitro* studies

Rabbit nucleus pulposus cells (rNPCs) culture was performed to characterize the biocompatibility of materials. Blend films were formed in 24-well cell culture plate as above procedures. These materials were irradiated with γ-ray before use. The rNPCs were provided by Second Affiliated Hospital of Soochow University, the density of rNPCs seeded on films was 2.0 × 10^6^/ml. The cell-seeded films were incubated for 4 hours to allow the cells to attach to the surface of the films, then a certain volume of Dulbecco's Modified Eagle Medium (H-DMEM) including 10% Fetal Bovine Serum (FBS) was added and continually cultured for 12 hours and 2 days. For the SEM observation, the cell-films after 12 hours and 2 days of culture were fixed for 6 hours with 2.5% glutaraldehyde, dehydrated in graded series of ethanol, dried and coated with gold, finally were examined by SEM.

## Results and discussion

### Morphology

**Fig. 2:**
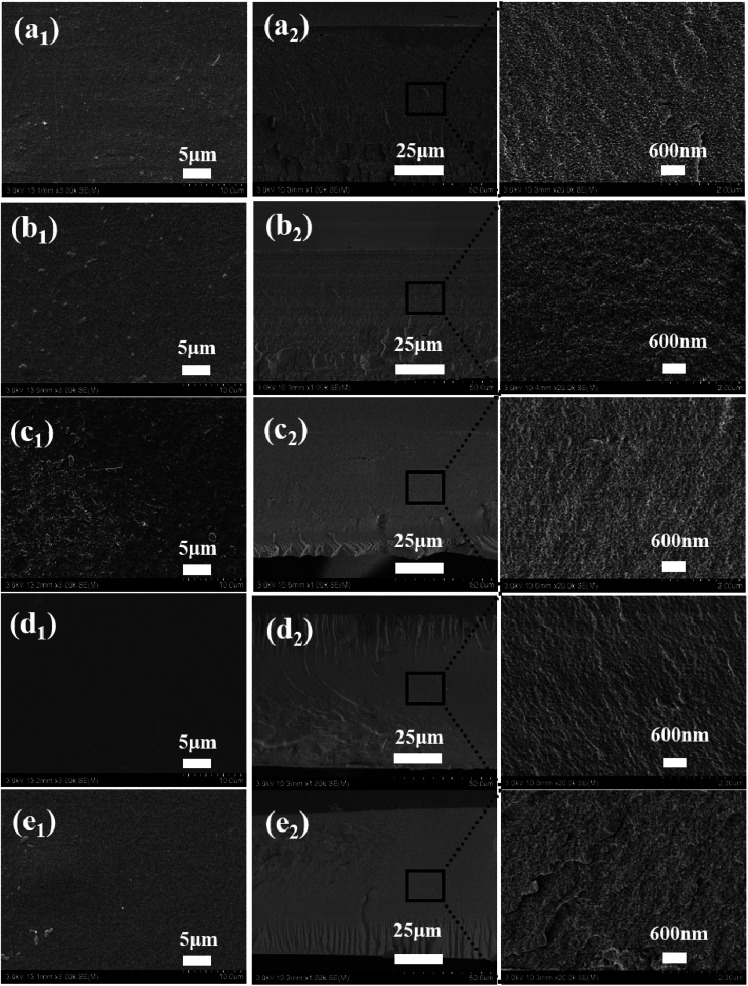
Surface (a_1_, b_1_, c_1_, d_1_ and e_1_) and cross-section morphology (a_2_, b_2_, c_2_, d_2_ and e_2_) of BSF/TSF blend films with various blend ratios. (a_1_ and a_2_) BSF, (b_1_ and b_2_) 3/1, (c_1_ and c_2_) 1/1, (d_1_ and d_2_) 1/3, (e_1_ and e_2_) TSF.

The surface and cross-sectional morphology of the BSF/TSF blend films were observed with scanning electron microscopy, as shown in Fig. 2. Overall, the blend films showed relatively flat surface as expected, indicating the good film forming property of fibroin-CaCl_2_/formic acid solution. However, close observation inside the films demonstrated different microstructure. The special nanostructure of BSF film was studied in our previous study.^23^ The BSF film showed nanofibrous structure originated from the nanofibril-preservation dissolution behavior of fibroin in CaCl_2_/formic acid. The preserved nanofibril endowed the BSF film with exceptional mechanical properties. Different with BSF film, the TSF film showed layer structure as previous reports.^24,25^ The specific nanostructure of BSF existed, and the layer structure of TSF disappeared in the blend films. Furthermore, the morphological phase separation was not observed, which had been reported in the BSF/TSF,^26^ and in the TSF/carboxymethyl chitosan blend films prepared using water as a co-solvent.^27^ The possible reason was that formic acid was an excellent solvent for silk fibroin dissolution and regeneration,^28^ but water was just a temporary solvent for silk fibroin in which gel formed easily.^29^ The sol–gel transition of TSF was more faster than that of BSF,^30^ which easily resulted in the phase separation in the blend films. Instead, the uniformly distribution of BSF and TSF in formic acid favored the formation of compatible BSF/TSF blend film. Therefore, CaCl_2_/formic acid was an excellent solvent for the dissolution and regeneration of BSF, TSF, and their hybrid, the resulting blend films showed smooth surface and specific nanostructure, as well as the compatibility of BSF and TSF.

### Structure characteristic

#### FTIR

**Fig. 3:**
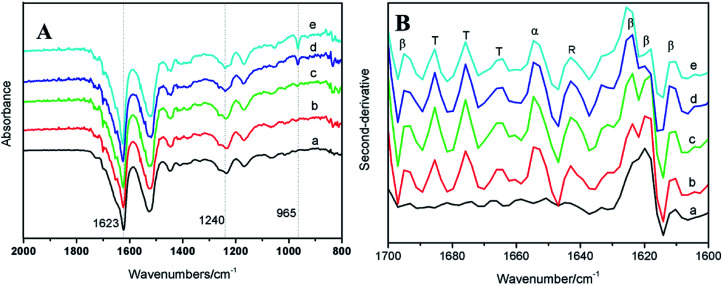
FTIR original spectra (A) and second-derivative spectra (B) of BSF/TSF blend films with various blend ratios. (a) BSF, (b) 3/1, (c) 1/1, (d) 1/3 and (e) TSF. The peaks were assigned as random coil (R), α-helix (α), β-sheet (β) and turn (T).

FTIR was a widely used technique to analyze the molecular conformation of silk fibroin because the amide bands of IR spectra were known sensitive to the molecular change of silk fibroin.^31^ The FTIR spectra of BSF/TSF blend films was shown in Fig. 3A. In addition to the characteristic peaks of β-sheet conformation at 1623 cm^−1^ (amide I), 1527 cm^−1^ (amide II) and 1265 cm^−1^ (amide III), the appearance of peak at 1240 cm^−1^ (amine III) was attributed to the random coil conformation,^32^ suggesting the predominantly β-sheet but with certain amount of random coil structure in water rinsed BSF film. In the spectra of TSF, the strong absorption bands at 1622, 1520, 1240 and 965 cm^−1^ supported the mainly β-sheet structure.^33,34^ The spectra of BSF/TSF blend films revealed that there was a weak, or even absent molecular interactions between the two fibroins because no obvious shift of absorption bands or appearance of new absorption bands was observed. To further confirmed the result, the second-derivative procedures was used to distinguish individual overlapping absorptions.^35^Fig. 3B showed the second-derivative spectra of amide I region of BSF/TSF blend films. Obviously, the numbers and positions of peaks were not changed, providing strong evidence that the secondary structure of BSF and TSF was not significantly by the blending of two fibroin, which was in good agreement with previous reports.^36,37^

#### X-ray diffraction curves

**Fig. 4:**
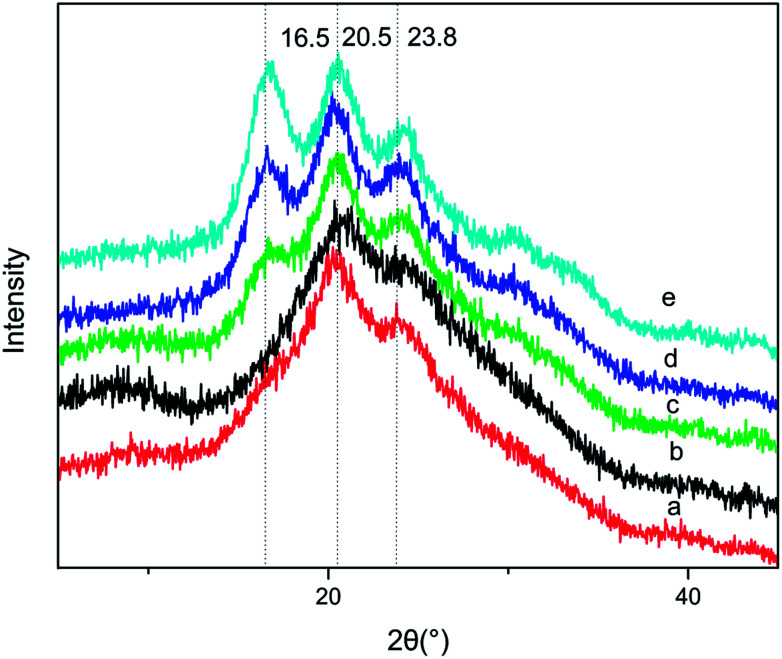
XRD spectra of BSF/TSF blend films with various blend ratios. (a) BSF, (b) 3/1, (c) 1/1, (d) 1/3, (e) TSF.

X-ray diffraction was carried out to study the crystalline structure of BSF/TSF blend films. Fig. 4 showed the X-ray diffraction curves of blend films with different BSF/TSF volume ratio, the pure BSF film was characterized by diffraction peak at 2*θ* values of 20.3° and 23.8°, corresponding to the spacing of 4.26 Å and 3.73 Å, respectively, attributing to β-sheet.^27^ The pure TSF showed diffraction peaks at 16.5°, 20.5° and 24.5° corresponding to the spacing of 5.37 Å, 4.33 Å and 3.63 Å, attributing to β-sheet.^38^ From the IR analysis, we knew that the molecular interaction between BSF and TSF was absent or very weak. Consistent with these results, the diffraction of the blend films (b–d) exhibited diffraction peaks' characteristic of BSF and TSF simply overlapping in Fig. 4.

However, the diffraction peak at 16.5° characteristic of TSF disappeared when the BSF content was up to 75%, demonstrating that the blending of BSF/TSF had affected the crystal structure of TSF. The low concentration of TSF may reduce the probability of TSF chains' aggregation and β-sheet conformation. In FTIR spectra, the absorption peak at 965 cm^−1^ attributed to the β-sheet was hard to be observed when the BSF content was 75%.

### Thermal analysis

#### TGA

**Fig. 5:**
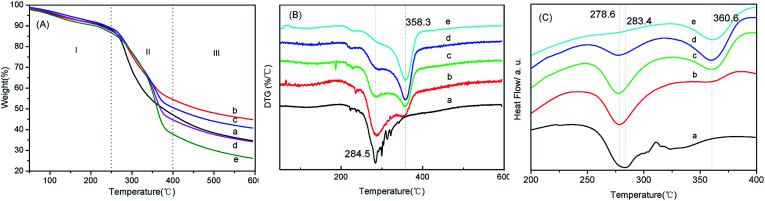
TGA (A), DTG (B) and DSC (C) curves of BSF/TSF blend films with various blend ratios. (a) BSF, (b) 3/1, (c) 1/1, (d) 1/3, (e) TSF, nitrogen flux at 30 ml min^−1^, heating rate at 10 °C min^−1^ and temperature range from 50 to 600 °C, and the samples weight was about 5 mg.

TGA was widely used technique to characterize silk fibroin^39^ and other polymers.^40^Fig. 5 showed the thermogravimetric curves of BSF/TSF blend films, which demonstrated the weight a continue weight loss (WL) to 600 °C. Overall, the weight decrease behavior in TGA curves can be divided into three distinct regions. Region I, the films showed a little and slow weight loss with the temperature increased up to approximately 250 °C. The first weight loss about 10% was mainly due to the water evaporation. Region II, an approximate linear TGA curve was observed in range of 267–383 °C, in which the weight loss decreased rapidly with temperature, ascribed to the breakdown of intermolecular interaction, as well as the cleavage of peptide bonds. Region III, the film demonstrated continue decomposition but slow weight loss behavior.

#### DTG

As shown in Fig. 5, BSF and TSF films showed different thermal decomposition peaks at 284.5 °C and 358.3 °C, respectively. As expected, the blend films showed two decomposition peaks which was accurately corresponding to the decomposition of BSF and TSF, respectively. It illustrated that the two silk fibroins didn't form co-crystal structure, as reported previously.^41^ The thermal decomposition process of blend films was composed of two stages: BSF and TSF thermal decomposition. They had two different decomposition peak temperature near 300 °C and 360 °C, respectively, which were decided by their different crystal structure.

#### DSC

DSC thermograms of BSF/TSF blend films were shown in Fig. 5C. The pure BSF film showed an endothermic peak at 284.5 °C without any trace of exothermic transition, and pure TSF film showed an endothermic peak at 360.6 °C, this behavior was due to β-sheet structure.^42,43^ In addition, BSF/TSF blend films appeared two endothermic peaks at 278.6 °C and 360.6 °C, which belonged to endothermic peaks of BSF and TSF, respectively. The result confirmed that BSF/TSF blend films were co-existence and did not appear co-crystallization. DSC experiment did not evidence phase transitions, suggesting the crystalline structure of silk fibroin consistent with above XRD and IR results.

The dissimilarities in the thermal behavior of BSF and TSF could be straightforwardly interpreted by their different crystalline structure. The amino acid sequence in the crystalline region of BSF and TSF was considered to be (GAGAGS)n (G: glycine, A: alanine, S: serine) and –(ala)–, respectively.^44,45^ So the TSF molecular chains would form a more compact crystalline structure than BSF, resulting in higher thermal decomposition peaks (Fig. 5).^46^ The *T*_d_ for BSF and TSF were 284 °C and 358 °C respectively. The *T*_d_ of BSF was comparable to those of other biodegradable polymers like cellulose (*T*_d_ = 266 °C), poly(hydroxy butyrate-valerate) (*T*_d_ = 287 °C), polypropylene (*T*_d_ = 295 °C) and polystyrene (*T*_d_ = 270 °C),^47^ all which were lower than that of TSF. As expected, the blending of TSF improved the *T*_d_ of BSF, in turn, the adding of BSF decreased the *T*_d_ of TSF (Table 1). But to our surprise, compared to the pure BSF and TSF, the residual masses of BSF/TSF blend films at 600 °C were all significantly higher than that of pure BSF and TSF films as previous report,^37^ suggesting that the thermal stability of silk fibroin could be improved by blending two different silk fibroin. The underlying mechanism was unclear, possibly due to the interaction between BSF and TSF that was not evidenced here and needed further study.

**Table 1:** Thermogravimetric parameters for BSF/TSF blend films obtained from the TG and DTG curves

	ML_120_ (wt%)	*T*_d_-BSF (°C)	*T*_d_-TSF (°C)	MR_600_ (wt%)
BSF	5.0	284.5		34.7
BSF/TSF (3/1)	5.2	287.9	350.7	44.7
BSF/TSF (1/1)	5.0	289.6	354.8	40.7
BSF/TSF (1/3)	4.8	291.4	356.6	34
TSF	5.9		358.3	26.1

### Mechanical properties

**Fig. 6:**
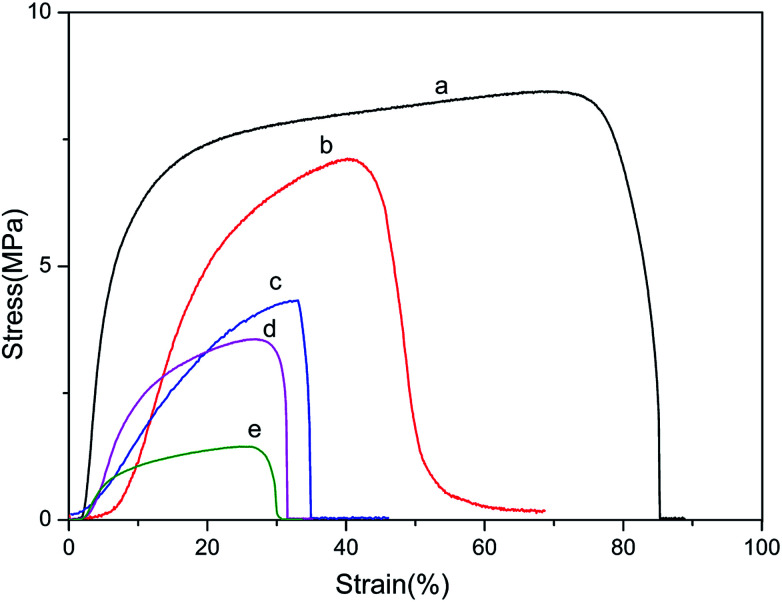
Stress–strain curves of BSF/TSF blend films with various blend ratios under wet conditions. (a) BSF, (b) 3/1, (c) 1/1, (d) 1/3 and (e) TSF.

Mechanical properties were of primary essential for determining the performance of materials expected to undergo various types of stresses during use. The representative stress–stain curves were presented in Fig. 6 and summaries of mechanical properties were listed in Table 2. The mechanical properties of blend films were obtained in the wet state, which was more important in practical applications of biomaterials than the dry state.

**Table 2:** Results of tensile test of obtained films under wet condition (*n* ≥ 5)

Film codes	Breaking stress (MPa)	Breaking strain (%)	Young's modulus (MPa)	Contact angle (°)
a	7.28 ± 0.83	73.54 ± 8.37	33.63 ± 0.80	76.04 ± 1.68
b	5.71 ± 0.30	46.76 ± 2.86	13.95 ± 0.78	74.86 ± 1.23
c	4.21 ± 0.27	28.76 ± 1.83	2.71 ± 0.51	71.04 ± 1.49
d	3.95 ± 0.25	26.80 ± 0.51	2.63 ± 0.25	64.99 ± 1.08
e	1.17 ± 0.13	21.70 ± 2.21	2.19 ± 0.83	64.11 ± 2.74

It can be found that wet film of BSF (a) was stronger and tougher, while pure TSF (e) was weak and fragile. In addition, the Young's modulus showed huge difference between different content of BSF in the blend films, the Young's modulus of pure BSF film was 33.63 ± 0.80 MPa, and the pure TSF was only 2.19 ± 0.83 MPa. From the Fig. 6 and Table 2, we also found that the breaking stress, breaking strain and Young's modulus of BSF/TSF blend films were continuously increasing along with the proportion of BSF, exhibiting the typical features of a miscible multi-component polymer material. This result also confirmed that TSF film had a poor mechanical properties.^46^

### Contact angles

To investigate the hydrophilicity of BSF, TSF and BSF/TSF blend films, water contact angle measurement was shown in Table 2. Smaller contact angle usually indicated that the material surface was more hydrophilic, enhancing the cell adhesion and proliferation.^48^Table 2 showed that the pure BSF film had the biggest contact angle, when TSF contents were 25%, 50% and 75% in the blend films, the contact angle decreased from 76.04 ± 1.68° to 74.86 ± 1.23°, 71.04 ± 1.49° and 64.99 ± 1.08°, respectively. It indicated that the introduction of TSF improved the hydrophilicity of BSF. Pure TSF film showed the smallest contact angle of 64.11 ± 2.74°, indicating the better hydrophilic property of TSF due to its high content of polar amino acid.^49^

### Biocompatibility

**Fig. 7:**
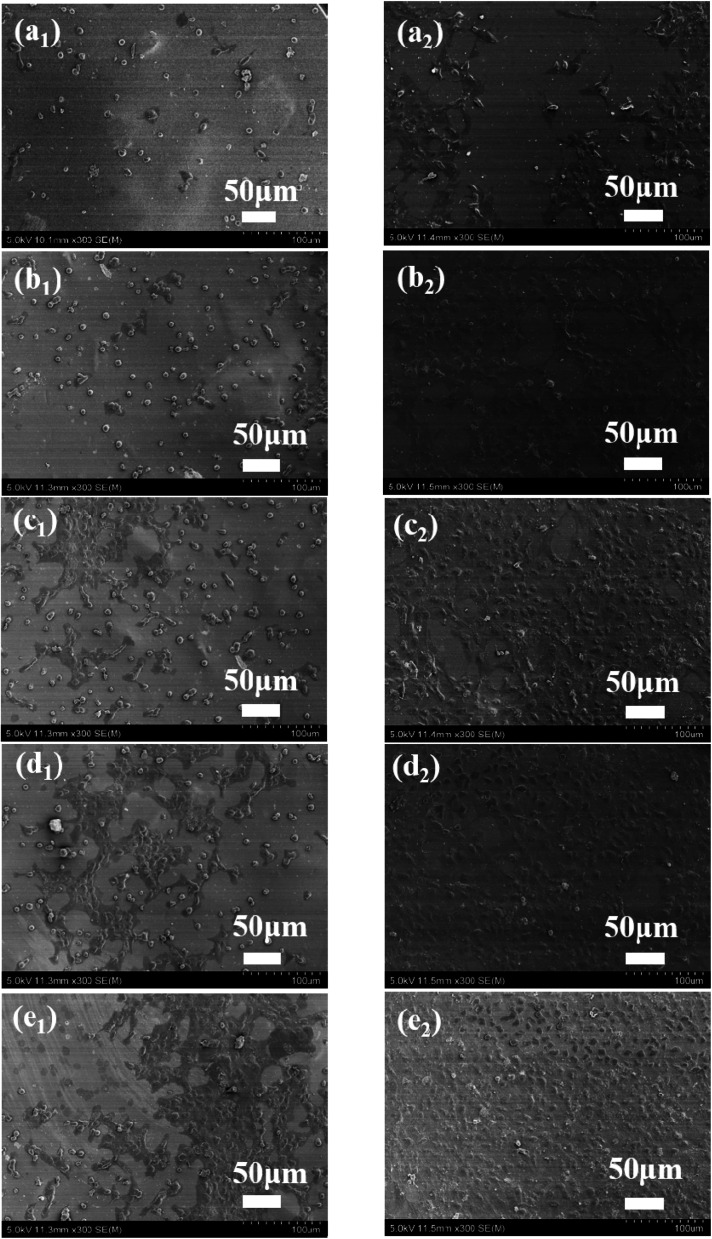
SEM of attachment and proliferation of rNPCs grew on BSF/TSF blend films with various blend ratios. (a_1_ and a_2_) BSF, (b_1_ and b_2_) 3/1, (c_1_ and c_2_) 1/1, (d_1_ and d_2_) 1/3, (e_1_ and e_2_) TSF. Culture time (a_1_, b_1_, c_1_, d_1_ and e_1_), (a_2_, b_2_, c_2_, d_2_ and e_2_) were 12 hours and 2 days, respectively.

To evaluate biocompatibility to the BSF, TSF and BSF/TSF blend films at different ratios, rNPCs were cultured on films. The long-term differentiation and stability were affected by initial attachment. Therefore, the adhesion and proliferation of rNPCs on films were examined by SEM. As shown in Fig. 7, the rNPCs grew in clusters, from the center to the surrounding area. When the culture period was 12 hours, more rNPCs can be observed on the surface of the pure TSF films comparing with pure BSF films. Toward the BSF/TSF blend films, with the increase of TSF, more rNPCs attached on the blend films. In addition, the morphology of rNPCs on pure BSF and BSF/TSF at ratio of 3/1 were granular, while rNPCs on the pure TSF, BSF/TSF blend films at ratio of 1/1 and 3/1 began to grow from center to the surrounding area. When the culture time was 2 days, rNPCs began to spread growth. Although the number of rNPCs observed on all the films increased from 12 hours to 2 days, with the increase of TSF content, rNPCs showed a better adhesion and proliferation properties. Which due to the TSF had more polar amino acids with positive charge and RGD sequence can support the cell growth that were absent from BSF, so the TSF showed a better compatibility comparing with BSF.

## Conclusions

In summary, we provided a novel method for the preparation of TSF/BSF blend films using CaCl_2_/formic acid as a co-solvent and water as post-treatment solvent. We showed that BSF and TSF was compatible in the BSF/TSF blend films because CaCl_2_/formic acid was an excellent solvent for BSF and TSF. The BSF/TSF blend films showed smooth surface and specific nanostructure in the cross-section. Water was a useful post-treatment solvent in regenerating stable silk fibroin films through inducing the formation of β-sheet. The direct use of water as a post-treatment solvent for CaCl_2_/formic acid dissolved silk fibroin suggested that the dissolution behavior was different to the traditional dissolution method, since traditionally derived BSF and TSF was water-soluble. Interestingly, the BSF/TSF blend films showed higher thermal stability compared to pure BSF and TSF film, but no obvious molecular interaction was detected through FTIR and XRD analysis. In addition, the good mechanical properties in wet state, controllable hydrophilicity, as well as excellent biocompatibility of the pure BSF, TSF and their hybrid films were also demonstrated. Thus, this study provided a useful method of preparing silk fibroin-based material for potential application in biomedical and biotechnological area.

## Conflicts of interest

There are no conflicts to declare.

## Supplementary Material
